# The Prognostic Significance of Cancer-Associated Fibroblasts in Esophageal Squamous Cell Carcinoma

**DOI:** 10.1371/journal.pone.0099955

**Published:** 2014-06-19

**Authors:** Sang Yun Ha, So-Young Yeo, Yan-hiua Xuan, Seok-Hyung Kim

**Affiliations:** 1 Department of Pathology, Samsung Medical Center, Sungkyunkwan University School of Medicine, Seoul, Korea; 2 Department of Health Sciences and Technology, Samsung Advanced Institute for Health Sciences and Technology, Sungkyunkwan University, Seoul, Korea; 3 Key Laboratory of Natural Resources of the Changbai Mountain and Functional Molecules, Ministry of Education, Yanbian University, Yanji, China; 4 Department of Pathology, Yanbian University School of Medicine, Yanji, China; Deutsches Krebsforschungszentrum, Germany

## Abstract

**Background:**

Cancer-associated fibroblasts (CAF) are activated fibroblasts in the cancer stroma and play an important role in cancer progression. Some reports have indicated the correlation between the expression of CAF markers and adverse prognosis in several cancers. However, no reports have studied CAF phenotype and its clinical relevance in esophageal squamous cell carcinoma (ESCC).

**Methods:**

We investigated CAF phenotype of ESCC based on histology and immunohistochemical expressions of five CAF markers such as fibroblast activation protein (FAP), smooth muscle actin (SMA), fibroblast-specific protein-1 (FSP1), platelet-derived growth factor receptor (PDGFRα), and PDGFRβ in 116 ESCC tissue samples. Besides, we also examined the correlation of the CAF phenotype with clinical relevance as well as other cancer-microenvironment related factors.

**Results:**

Histologically immature CAF phenotype was correlated with poor prognosis (p<0.001) and associated with increased microvessel density, increased tumor associated macrophages, and epithelial to mesenchymal transition. CAF markers were characteristically expressed in stromal fibroblast close to tumor cells and the expression pattern of 5 CAF markers was highly heterogeneous in every individual cases. Of five CAF markers, SMA, FSP1, and PDGFRα were unfavorable prognostic indicators of ESCC. The number of positive CAF markers was greater in ESCC with immature CAFs than in those with mature ones.

**Conclusions:**

Our results demonstrate that histologic classification of CAF phenotype is a reliable and significant prognostic predictor in ESCC. CAF markers have the potential to be diagnostic and therapeutic targets in ESCC.

## Introduction

Cancer-associated fibroblasts (CAFs) are activated fibroblasts in the cancer stroma and are at the leading edge of many solid tumors, including breast, colon, and melanoma.[1]–[5] CAFs are the most prominent cell type within the tumor stroma of many cancers and an important player in the cancer-microenvironment which consists of a dynamic mixture of fibroblasts, monocytes/macrophages, endothelial cells, lymphocytes, and granulocytes.[2], [6] CAFs drive tumor progression by directly stimulating tumor cell proliferation through the secretion of various growth factors and cytokines such as hepatocyte growth factor, transforming growth factor-β, stromal cell-derived factor 1, and interleukin-6 as well as by remodeling the cancer microenvironment through deposition of extracellular matrix and recruitment of other players such as various inflammatory cells and endothelial cells.[1]–[3] Furthermore, activated CAFs contribute to cancer progression by inducing angiogenesis.[1]–[3] During cancer progression, CAFs contribute to invasive growth of cancer by secreting several proteases such as matrix metalloproteinase or cathepsins and inducing the epithelial to mesenchymal transition (EMT) [1]–[3].

Although fibroblasts are widely distributed and easily recognizable due to their fusiform or spindle-like shape, fibroblasts remain poorly defined in molecular terms and there is no known specific and reliable molecular fibroblast markers.[4] Therefore, the identity of CAF is also poorly understood and CAF marker with absolute specificity has not been identified. There are several well-established indicators of CAF such as smooth muscle actin (SMA), fibroblast stimulating protein-1 (FSP-1), platelet-derived growth factor α (PDGFRα), and PDGFRβ.[4] However, none of them are both exclusive to CAFs and present in all CAFs.[4] Instead, each of these CAF markers is estimated to represent a certain and different phenotype of CAFs. In addition, fibroblasts are highly heterogeneous and fibroblasts from different anatomical sites have considerably different expression profile [7].

In general, CAFs have been known to have distinct morphology characterized as large and plump cells distinguished from normal fibroblasts which are thin, wavy, and small spindle cells.[4], [6], [8] However, we have preliminarily found that some ESCC had stromal fibroblasts having histology of relatively normal fibroblast, while some had distinct morphology of CAF.

The classification by histological morphology of individual CAF and its clinical relevance have not been studied, although some previous reports showed that histological categorization of stromal fibrosis was correlated with clinical outcome of colon cancer [9], breast cancer [10], and lung cancer [11].

Esophageal squamous cell carcinoma (ESCC) is one of the most aggressive malignant tumors, with a 5-year survival rate of only 10%.[12] CAF have seldom been discussed in ESCC despite its significance in cancer progression. Only very few studies have investigated the biological role of the tumor stroma including angiogenesis in ESCC.[13]–[18] Because fibroblasts in different parts of the body are intrinsically different, the result of CAF study in cancer of different organs cannot be directly applied to ESCC.

Therefore, we studied clinical relevance of CAF in ESCC by histological classification according to individual cell morphology and immunohistochemical studies of five CAF markers such as FAP, SMA, FSP-1, PDGFRα, PDGFRβ. In addition, we assessed the association between CAF and other cancer-microenvironment related factors such as microvessel density (MVD) and tumor associated macrophage (TAM) infiltration or EMT phenotype.

## Materials and Methods

### Tissue Specimens

A total 116 formalin-fixed and paraffin-embedded tumor samples from patients who underwent curative surgical resection for primary ESCC at the Samsung Medical Center, Seoul, Korea from 1995 to 2008, were included. This retrospective study was approved by institutional review board at Samsung Medical Center, and conducted in accordance with the 1996 Declaration of Helsinki. All patients provided written informed consent according to institutional guidelines. No patient received preoperative chemotherapy or radiotherapy. Postoperative adjuvant treatment was performed in 86.2% (100/116) of the patients: chemotherapy and radiotherapy in 31 patients, chemotherapy only in 51 patients, and radiotherapy only in 18 patients. Clinical and pathological reports were reviewed for age, sex, tumor size, histological grade, invasion depth (pT), nodal status (pN), and distant metastasis (pM). The median follow-up period was 30 months (range 0–108 months). The pTNM classification was applied according to guidelines from the 2010 American Joint Committee on Cancer staging manual (AJCC 7^th^ edition).

### Tissue Microarray (TMA) Reconstruction

Hematoxylin and eosin (HE)-stained tissues were reviewed to confirm the histological diagnosis and to select representative areas for immunostaining. One or two cylindrical core (2 mm in diameter) was removed from formalin-fixed and paraffin-embedded tissue blocks corresponding to the HE slides to construct the tissue microarray. Each core was selected to contain both tumoral (20–50%) and stromal (50–80%) component. To minimize selection bias, each tissue core was carefully chosen to contain at least one or more CAF’s “hot spot” crowded with CAFs and stromal cells. Sectioning of microarray blocks produced 4 mm thick sections after completion of the tissue array.

### Classification of CAFs by Histology on Hematoxylin and Eosin (HE) Slides

**Figure 1:**
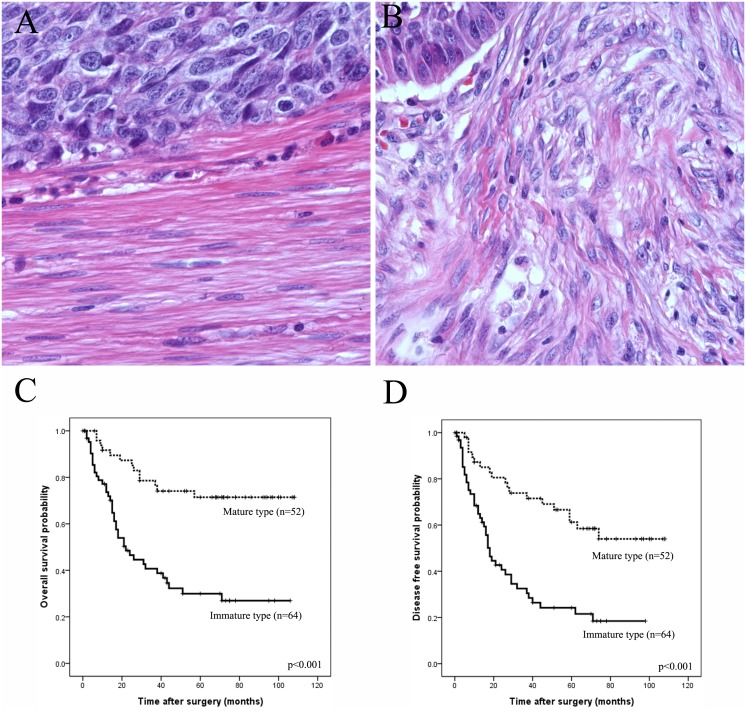
Histological categorization of stromal fibroblast on hematoxylin and eosin slides. A. Mature type when fibroblasts show thin, wavy, and small spindle cell morphology; normal fibroblasts; B. Immature type when fibroblasts show large, plump spindle-shaped morphology; C–D. Survival curves using the Kaplan–Meier method by log-rank test for histologic subtype of cancer associated fibroblast.

CAFs were divided into two groups according to their morphology on HE slides, by two experienced pathologists (YHX and SHK) with no prior knowledge of clinicopathological results, as below: 1. mature when fibroblasts showed thin, wavy, and small spindle cell morphology as normal fibroblasts; 2. Immature when fibroblasts showed large, plump spindle-shaped cell with prominent nucleoli (Figure 1). When the proportion of immature fibroblasts was more than 50%, the case was regarded as having immature CAF phenotype. In a few cases with disagreement, final interpretation was determined by consensus using the multi-head microscope.

### Immunohistochemical Staining Procedure

Sections on microslides were deparaffinized with xylene, hydrated using a diluted alcohol series, and immersed in 0.3% H_2_O_2_ in methanol to quench endogenous peroxidase activity. Sections were treated with TE buffer (10 mM Tris and 1 mM EDTA, pH 9.3) at 98°C for 30 min. To reduce non-specific staining, each section was blocked with 4% bovine serum albumin in PBS with 0.1% Tween 20 for 30 min. The sections were then incubated with anti- SMA (1∶100, Millipore, Billerica, MA, USA), anti-FAP (1∶100, Abcam), anti-FSP1 (1∶100, Millipore), anti-PDGFRα (1∶100, Cell Signaling Technology), anti-PDGFRβ (1∶100, Abcam), anti-CD34 (1∶100, Dako, Glostrup, Denmark) and anti-CD68 (1∶1000, Dako) in PBST containing 3 mg/ml goat globulin (Sigma, St. Louis, MO, USA) for 60 min at room temperature, followed by three successive washes with buffer. Sections were then incubated with an anti-mouse/rabbit antibody (Envision plus, Dako) for 30 min at room temperature. The chromogen used was 3,3′-diaminobenzidine (Dako). Sections were counterstained with Meyer’s hematoxylin. Omitting the primary antibody provided negative controls for immunostaining.

### Evaluation of the Immunohistochemical Analysis

**Figure 2:**
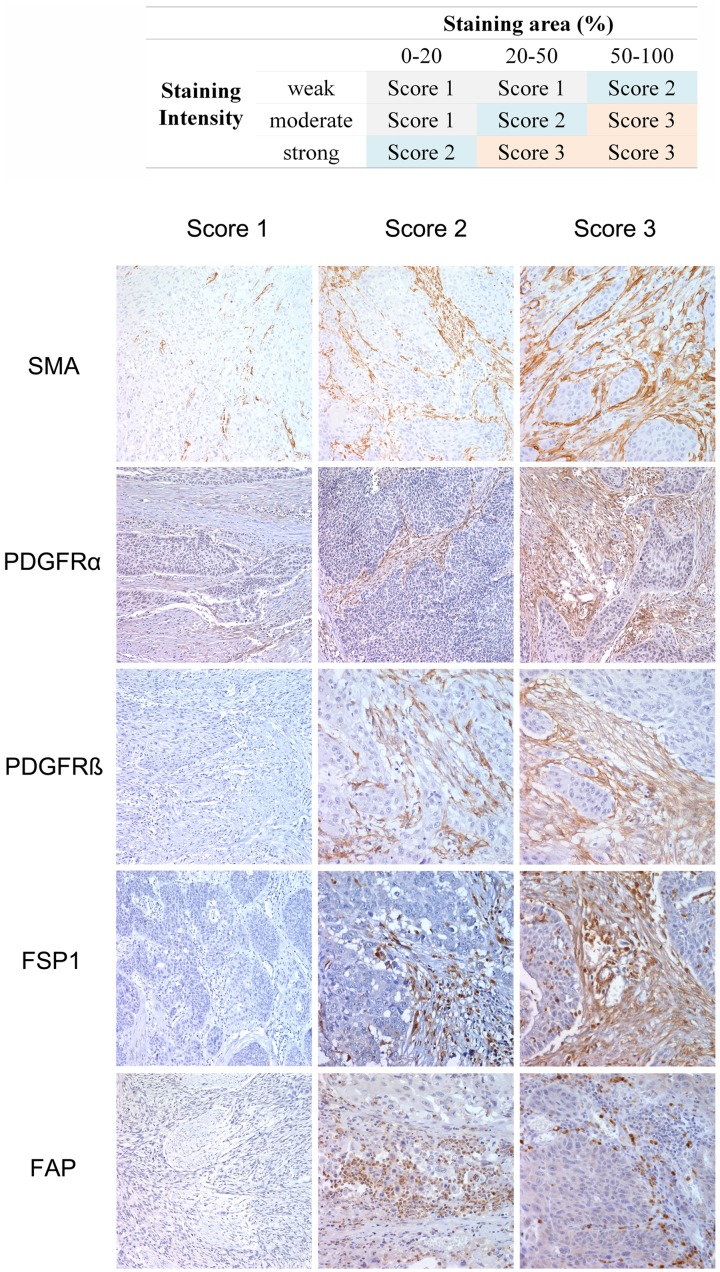
Immunohistochemical staining of cancer-associated fibroblast related fibroblast markers. A: Analysis of immunohistochemical staining performed based on both intensity and staining area. B: Demonstration of the immunohistochemical staining of smooth muscle actin (SMA), fibroblast-specific protein-1 (FSP-1), fibroblast activator protein (FAP), platelet-derived growth factor receptor (PDGFR)α, and PDGFRβ.

Two pathologists (YHX and SHK) evaluated the immunohistochemical results with no prior knowledge of clinicopathological results, and discussed any discrepancies in scores until a consensus was reached. According to the staining intensity and the proportion of positive stromal cells, immunohistochemical scores for SMA, FAP, FSP1, PDGFRα, and PDGFRβ were measured by two pathologists (YHX and SHK) with no prior knowledge of clinicopathological results, as follows: 1, weak staining in <50% or moderate staining in <20% of stromal cells; 2, weak staining in ≥50%, moderate staining in 20–50% or strong staining in <20%; 3, moderate staining in ≥50% or strong staining in ≥20% (summarized in Figure 2). Cases with score 2 and 3 were regarded as positivity for each protein expression. MVD was evaluated by method of Weidner et al.[19] Briefly, all CD34 positive individual microvessel counts were made on a 200× field after the highest area was identified by scanning at low magnification (40–100×). MVD was classified into three groups: low when MVD was <40; intermediate when MVD was 40–60; and high when MVD was >60. TAM was identified by positivity of CD68 and the degree of TAM infiltration was determined by percentage of CD68 positive cells among all stromal cells: low when percentage was <20%; high when ≥20% [20].

### Statistical Analysis

Correlations were examined using Pearson’s chi-square test or Fisher’s exact test as appropriate. Overall survival (OS) and disease free survival (DFS) were determined using the Kaplan–Meier method and were compared using the log-rank test. Survival was measured from the date of surgery. The Cox proportional hazards model was used for multivariate analysis. Cinicopathologic factors, which were statistically significant in univariated analysis, were included as covariables in multivariate analysis. Hazard ratios (HR) and 95% confidence intervals (CI) were assessed for each factor. All tests were two sided, and *P*≤0.05 was considered significant. The statistical analysis was performed using SPSS statistical software (SPSS Inc, Chicago, IL, USA).

## Results

### Histological Classification of CAF is Correlated with Prognosis in ESCC

Fifty two cases (44.8%) were classified as mature CAF phenotype and 64 ones (55.2%) as immature phenotype (Figure 1A–B). The correlations between these categories and the clinicopathologic features are summarized in Table 1. The immature CAF phenotypes were significantly associated with increased MVD (p<0.001) and enhanced infiltration of TAM (p = 0.003) compared to the mature stromal phenotype. Both increased MVD and infiltration of TAM are correlated with poor prognosis of ESCC (Figure S1). This histological categorization was also associated with the EMT phenotype of ESCC (p = 0.002), which was defined in our previous study by immunohistochemical expression of mesenchymal marker and E-cadherin.[21] The complete EMT phenotype, characterized by loss of E-cadherin expression with acquirement of mesenchymal marker expression, was significantly more frequent in the immature CAF phenotypes than that in the other phenotypes. The immature CAF phenotype was strongly correlated with decreased OS and DFS (p<0.001) (Figure 1C–D), and was a strong independent prognostic factor for OS (HR: 5.23 (95% CI: 2.57–10.65), p<0.001) and DFS (HR: 3.05 (95% CI: 1.66–5.59), p<0.001) (Table 2).

**Table 1:** Comparison of clinicopathologic characteristics according to histologic subtypes of cancer associated fibroblast.

	Cancer associated fibroblast (CAF) grade			
	Total	Mature	Immature	
	n = 116 (100.0)	52 (44.8%)	64 (55.2%)	p value
Age (years)				
<65	31 (26.7)	12 (23.1)	19 (29.7)	0.424
≥65	85 (73.3)	40 (76.9)	45 (70.3)	
Gender				
female	4 (3.4)	49 (94.2)	63 (98.4)	0.324^a^
male	112 (96.6)	3 (5.8)	1 (1.6)	
Tumor size (cm)				
<4	47 (40.5)	18 (34.6)	29 (45.3)	0.243
≥4	69 (59.5)	34 (65.4)	35 (54.7)	
Differentiation				
well	17 (14.7)	8 (15.4)	9 (14.1)	0.127
moderately	76 (65.5)	38 (73.1)	38 (59.4)	
poorly	23 (19.8)	6 (11.5)	17 (26.6)	
T stage				
1	15 (12.9)	4 (7.7)	11 (17.2)	0.239
2	40 (34.5)	21 (40.4)	19 (29.7)	
3	45 (38.8)	18 (34.6)	27 (42.2)	
4	16 (13.8)	9 (17.3)	7 (10.9)	
N stage^b^				
0	35 (32.1)	17 (35.4)	18 (29.5)	0.502
1	30 (27.5)	14 (29.2)	16 (26.2)	
2	26 (23.9)	12 (25.0)	14 (23.0)	
3	18 (16.5)	5 (10.4)	13 (21.3)	
M stage				
0	99 (85.3)	44 (44.4)	55 (55.6)	0.841
1	17 (14.7)	8 (47.1)	9 (52.9)	
Microvessel density^c^				
low	45 (39.1)	39 (75.0)	6 (9.5)	<0.001
intermediate	39 (33.9)	13 (25.0)	26 (41.3)	
high	31 (27.0)	0 (0)	31 (49.2)	
Tumor associatedmacrophages^c^				
low	50 (43.5)	31 (59.6)	19 (30.2)	0.002
high	65 (56.5)	21 (40.4)	44 (69.8)	
Epithelial to mesenchyaltransition				
complete	25 (21.6)	4 (7.7)	21 (32.8)	0.001
incomplete	31 (26.7)	20 (38.5)	11 (17.2)	
wild	60 (51.7)	28 (53.8)	32 (50.0)	

a by Fisher’s exact test, otherwise chi square test.

b Severn cases of unsatisfactory for minimal number of evaluated lymph nodes, were excluded in the analysis.

c One case was excluded in the analysis of microvessel density and macrophages due to lack of tissue.

**Table 2:** Multivariate Cox proportional hazard model analysis by classification of cancer associated fibroblasts.

Characteristic	Category	Overall survival			Disease-free survival		
		HR	95% CI	p-value	HR	95% CI	p-value
Differentiation	Well	1.00	Reference		1.00	Reference	
	Moderate	0.69	0.18–2.67	0.588	1.07	0.30–3.73	0.922
	Poor	2.15	0.54–8.59	0.281	1.70	0.47–6.21	0.420
T stage	1	1.00	Reference		1.00	Reference	
	2	10.53	1.26–88.06	0.030	3.16	0.83–12.06	0.092
	3	33.60	3.83–294.67	0.002	8.21	1.89–35.79	0.005
	4	28.25	2.89–276.53	0.004	7.16	1.54–33.36	0.012
N stage^a^	0	1.00	Reference		1.00	Reference	
	1	0.27	0.08–0.87	0.029	0.56	0.20–1.59	0.279
	2	1.47	0.52–4.18	0.469	1.70	0.67–4.28	0.262
	3	2.22	0.77–6.43	0.140	2.27	0.86–6.00	0.098
M stage	0	1.00	Reference		1.00	Reference	
	1	1.38	0.55–3.47	0.498	1.45	0.65–3.25	0.371
Radiotherapy	Negative	1.00	Reference		1.00	Reference	
	Positive	1.61	0.85–3.04	0.142	2.08	1.21–3.58	0.008
Cancer associatedfibroblast	Mature	1.00	Reference		1.00	Reference	
	Immature	5.23	2.57–10.65	<0.001	3.05	1.66–5.59	<0.001
SMA expression	Negative	1.00	Reference		1.00	Reference	
	Positive	0.95	0.24–3.86	0.944	1.20	0.39–3.71	0.754
FSP1 expression	Negative	1.00	Reference		1.00	Reference	
	Positive	4.86	1.90–12.40	0.001	2.77	1.39–5.51	0.004
FAP expression^b^	Negative	1.00	Reference				
	Positive	1.64	0.86–3.15	0.136			
Tumor associatedmacrophages^c^	Low	1.00	Reference		1.00	Reference	
	High	1.17	0.56–2.43	0.680	1.09	0.79–1.49	0.598
Microvessel density^c^	Low	1.00	Reference		1.00	Reference	
	Intermediate or high	1.45	0.66–3.19	0.351	1.32	0.65–2.67	0.449
Epithelial to mesenhymaltransition	Wild or incomplete	1.00	Reference		1.00	Reference	
	Complete	1.34	0.69–2.63	0.388	1.47	0.77–2.82	0.241

a Seven cases of unsatisfactory for minimal number of evaluated lymph nodes, were excluded in the analysis.

b FAP expression was not significant in univariate analysis for disease-free survival.

c One case with lack of tissue was excluded in the analysis.

### Expressions of CAF Markers such as SMA, FSP1, FAP, PDGFRα, and PDGFRβ in Stromal Fibroblasts are Frequent in ESCC and SMA & FSP1 are Strongly Associated with Adverse Clinical Outcome

We validated antibody against FSP1, FAP, PDGFRα, and PDGFRβ, except SMA, widely used in daily practice (Figure S2 and Text S1) and performed subsequent IHC procedure. The association of expression of five CAF markers with the clinicopathological features is summarized in Table S1. SMA, FSP1, FAP, PDGFRα, and PDGFRβ were expressed in 82.8%, 72.4%, 61.2%, 88.8%, and 54.3% of stromal fibroblasts in patients with ESCC, respectively (Figure 2). SMA expression in cancer stromal fibroblast was observed more frequently in tumors whose size is more than 4 cm (p<0.001), advanced T stage (p<0.001) and N stage (p<0.001), but less frequently in those with well differentiated tumors (p<0.001). It was also associated with EMT phenotype (p = 0.005). FSP1 expression was more frequently found in older patients (≥65 years) (p = 0.037). PDGFRβ expression was associated with poorly differentiated tumors (p = 0.010). However there are no significant correlations between FAP, PDGFRα and clinicopathologic parameters.

**Figure 3:**
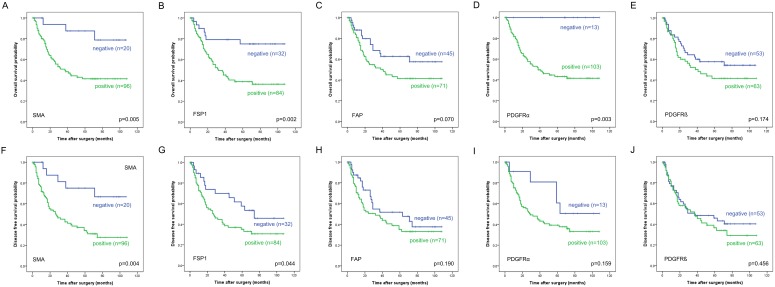
Overall and disease free survival curves using the Kaplan–Meier method by log-rank test for cancer associated fibroblast (CAF)-activation protein expression: (A, F) SMA; (B, G) FSP1; (C, H) FAP; (D, I) PDGFRα; (E, J) PDGFRβ.

The Kaplan Meier survival curves according to expression pattern of 5 CAF markers are provided in Figure 3. The expression of SMA and FSP1 was significantly correlated with shorter OS and DFS rates of patients. In particular, the 5-year OS and DFS rate of the stromal SMA-positive group (41% and 34% respectively) was significantly lower than those of the SMA-negative group (88% and 75%) (OS: p = 0.005; DFS: p = 0.004). The 5-year OS and DFS rates in the stromal FSP1-positive group (39% and 35% respectively) were also significantly lower than those in the of FSP1-negative group (79% and 58%) (OS: p = 0.002; DFS: p = 0.044). In addition, the 5-year OS rate in the stromal PDGFRα-positive group (43%) was significantly lower than that in the PDGFRα-negative group (100%) (p = 0.003). Patients with FAP expression showed shorter 5-year overall survival rate (41% vs 63%) without statistical significance (p = 0.070). And no significant association was observed between the PDGFRβ expression and OS or DFS rates. On multivariate analysis, FSP1 expression was an independent prognostic factor for OS (HR: 4.86 (95% CI: 1.90–12.40), p = 0.001) and DFS (HR: 2.77 (95% CI: 1.37–5.51), p = 0.004).

### The Correlation of CAF Marker Expression with Histological Classification

**Figure 4:**
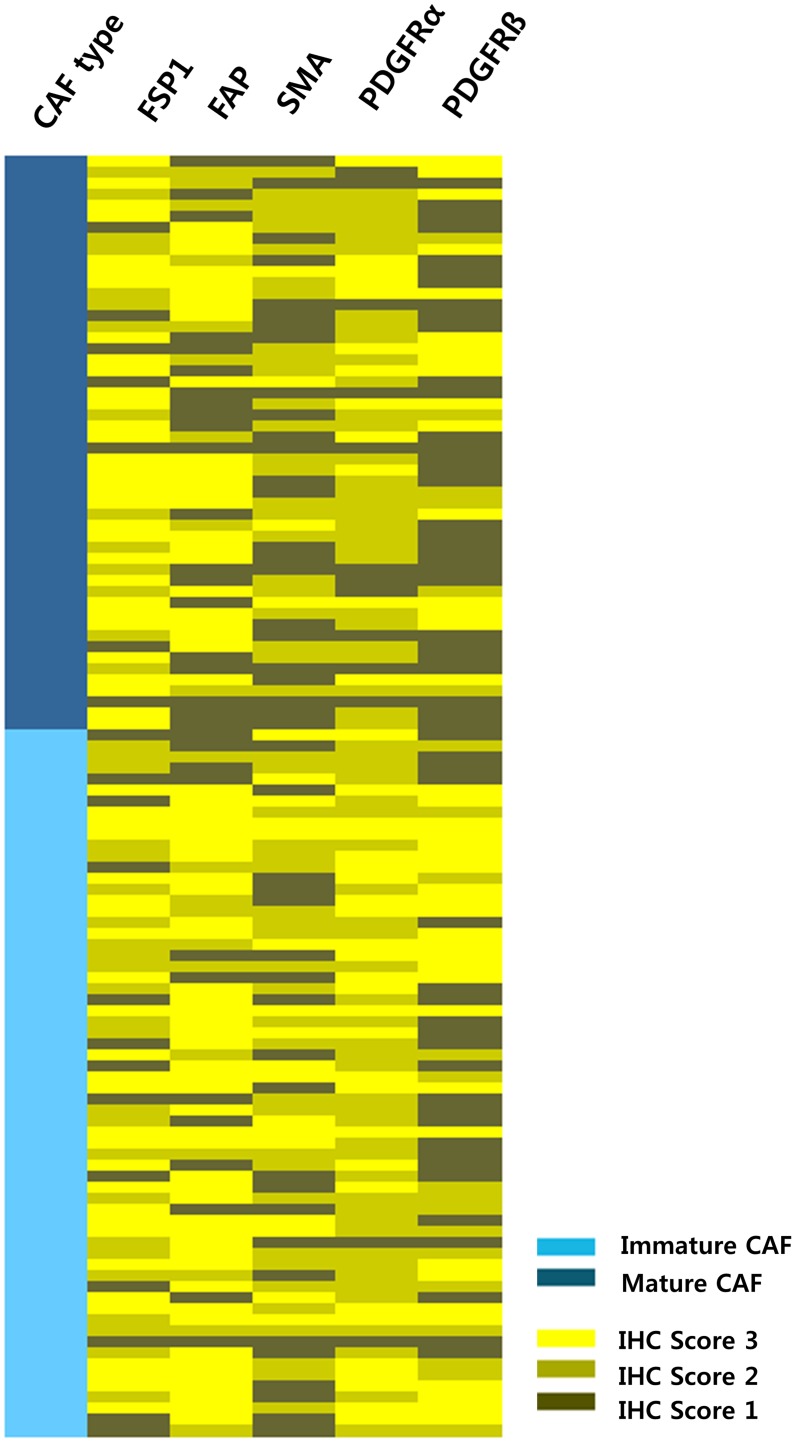
Heatmap of 116 esophageal squamous cell carcinoma cases according to histologic subtype and expression pattern of cancer associated fibroblasts.

Five CAF markers such as SMA, FSP1, FAP, PDGFRα, and PDGFRβ were heterogeneously expressed in stromal cells of 116 ESCCs (Figure 4). However, of these five CAF markers, the number of markers showing positivity in CAF was greater in ESCC with immature CAFs than them with mature ones (mean 3.89±1.09 vs 3.21±1.23, p = 0.002). Specifically, FSP1, PDGFRα or PDGFRβ expression was more frequently found in ESCCs with immature CAFs than them with mature ones (Table S2).

## Discussion

In this study, we first examined the expression pattern and clinical significance of CAF markers as well as the histological categories and other cancer-microenvironments in ESCC. Histologically immature CAF, defined as a large, plump spindle-shaped morphology, was associated with increased MVD, marked TAM and complete EMT phenotype. It was also a strong independent prognostic factor. Five CAF markers were heterogeneously expressed in individual case, but the total number of highly-expressed CAF markers was larger in immature CAF type than in mature one.

Fibroblasts seem to be the most versatile of connective-tissue cells, displaying a remarkable capacity to differentiate into other members of the family such as fat cells, cartilage cells, and bone cells. “Mature” fibroblasts with a lesser capacity for transformation may, for example, exist side by side with “immature” fibroblasts (often called mesenchymal cells) that can develop into a variety of mature cell types. Generally, immature fibroblasts are known to show typically a large plump and euchromatic nucleus with one or two nucleoli and rough endoplasmic reticulum and prominent Golgi apparatus on ultrastructural findings. In this study, we classified CAF of ESCC as a mature and immature type on the basis of histological features. As a result, immature type was observed in 55.2% of ESCC and was associated with adverse clinical outcome. Also it was associated with increased MVD and the complete EMT phenotype, which are known to be the mechanism of cancer progression.[1]–[6], [22] In addition, we noted the TAM as indicated by CD68 expression was significantly more frequent in ESCC with immature type CAF. Generally TAM is regarded as the M2 phenotype of macrophages that promote angiogenesis, growth, invasion, migration, and metastasis.[23]–[25] Infiltrating TAMs are correlated with poor prognosis in breast cancer.[23] In agreement with this report, enhanced infiltration of TAM in ESCC was also correlated with adverse clinical outcome. However, further study is required to determine more specific relationship between CAF phenotype and M2 phenotype of TAM in ESCC. These results suggest that our histological classification of stromal fibroblast is reliable and clinically relevant despite the lack of detailed understanding of its molecular mechanism.

We chose SMA, FSP-1, FAP, PDGFRα, and PDGFRβ as CAF markers based on previous studies.[1], [2], [4] This is the first study to investigate the expression and significance of SMA, FSP-1, FAP, PDGFRα, and PDGFRβ in ESCC as far as we know. Except SMA, which is widely used in daily practice, the remaining four antibodies are relatively new and have been used mostly for research purposes. Therefore we attempted to validate these four antibodies and found that all four antibodies were specific in both Western blotting and immunohistochemistry in formalin fixed and paraffin embedded tissue (Figure S2).

Our results revealed that SMA expression in ESCC stromal fibroblasts was associated with larger size, advanced T stage, lymph node metastasis, and poor prognosis. Despite the difference in organs and cancer types, this result is highly consistent with previous studies in which patients with abundant stromal myofibroblasts expressing SMA showed a poorer prognosis in oral, colorectal and breast cancers.[26]–[29] Our results also showed that patients expressing FSP1 were older and had shorter survival rates, which was also consistent with previous studies performed on breast cancer.[30] PDGFRβ expression was associated with poorly differentiated tumors (p = 0.010) but was not associated with prognosis. PDGFRα expression in CAF is an essential factor in the progression of lung cancer [31] and this result was partly consistent with our result. The patients with FAP expression showed slightly reduced OS (p = 0.070) and FAP expression was associated with more frequent death (p = 0.030). These results are partly in line with previous studies performed in pancreatic and colorectal cancers.[32], [33] Regarding the prognostic value of PDGFRβ, there is some disagreement between our result and those of previous two studies which remarked the association of stromal PDGFRβ expression and poor prognosis in prostatic and pancreatic cancer, respectively.[34], [35] This discrepancy may be attributable to different cancer types or organs. Our results demonstrated that some of individual CAF markers were significant prognostic predictors of ESCC. However, the expression pattern of CAF markers was highly heterogeneous in every individual case, reflecting the heterogeneity of CAF population. Considering the diversity of cellular origin of CAF and immense heterogeneity of CAF phenotypes, additional CAF markers with a good specificity are required [2].

Because of heterogeneity of CAF phenotype, the evaluation of CAF markers by tissue microarray (TMA) may have a potential limitation compared with evaluation by whole block-based immunostaining. However, we found that CAFs were evenly distributed irrespective of heterogeneity of CAF phenotype in more than half of ESCC cases (Figure S3A–B) in preliminary study examining expression of FSP1, a representative CAF marker, in the whole blocks of 10 ESCC cases. In the remaining cases, CAF distribution showed regional concentration around diverse location, such as invading front, surface necrosis or muscle layer (Figure S3C–E), without a specific correlation with anatomical location or depth of invasion. And CAF’s “hot spot” intensely crowded with CAFs and stromal cells was easily recognized on H&E section. To minimize selection bias, we used large-sized tissue core (2.0 mm in diameter) for TMA construction and each tissue core was selected to contain at least one or more CAF’s “hot spot”.

In ESCCs, recently several reports have shown the clinical significance and the role of tumor stroma including CAF in cancer progression. Wang et al. reported that the tumor-stroma ratio determined by microscopic evaluation was an independent predictor of survival in ESCC.[13] Liu et al. showed that the densities of myofibroblasts, lymphocytes, macrophages and microvessels were increased characteristically in the tumor stroma of ESCC and were usually associated with lymph node involvement.[14] Zhang et al. compared gene expression profiling of tumor fibroblasts from ESCC to that of normal fibroblasts and found that genes associated with cell proliferation, the extracellular matrix, and the immune response are differentially expressed.[16] These results are generally in line with our results in that the presence of activated CAFs was associated with an unfavorable outcome of ESCC. However, this is the first study that has evaluated the exact clinicopathological relevance of each CAF markers using a large cohort of patients with ESCC. In addition, this is the first study to analyze stromal fibroblasts in ESCC based on histology and show its prognostic effect in a large cohort of patients with ESCC.

In conclusion, our results demonstrate that histological classification of CAF is a powerful prognostic predictor for ESCC and is associated with increased MVD and TAM as well as complete EMT phenotype. Our results also suggest that CAF markers have potentials to be diagnostic and therapeutic targets in ESCC.

## Supporting Information

Figure S1 **Survival curves using the Kaplan–Meier method by log-rank test for cancer-microenvironment related factors.** (A–B) Microvessel density (C–D) Tumor associated macrophages.
(TIF)

Figure S2 **Validation of fibroblast activator protein (FAP), fibroblast-specific protein-1 (FSP-1), platelet-derived growth factor receptor (PDGFR)α and PDGFRβ antibodies.** A, B: The results of Western blotting and mRNA level by reverse transcription polymerase chain reaction. All antibodies recognized the proteins with the expected molecular weights (A) and these results were highly consistent with the mRNA levels of these proteins (B). C: The results of immunohistochemical staining of cell blocks from 293T, NIH3T3, and HeLa cells. Antibodies to FSP1, PDGFRα, and PDGFRβ stained in NIH3T3 cells (positive control) but not in 293T and Hela cells (negative control). Antibody to FAP also stained in Hela cells (positive control) but not in 293T or NIH3T3 cells (negative control).
(TIF)

Figure S3 **The result of preliminary study examining expression of FSP1, a representative cancer associated fibroblast (CAF) marker, in the whole blocks of 10 esophageal squamous cell carcinoma (ESCC) cases.** (A–B) CAFs are evenly distributed irrespective of heterogeneity of CAF phenotype in more than half of ESCC cases. (C–E) The remaining cases show CAF distribution with regional concentration around diverse location, such as invading front (C), surface necrosis (D) or muscle layer (E).
(TIF)

Table S1 **Comparison of clinicopathologic characteristics according to expression pattern of SMA, FSP1, FAP, PDGFRA and PDGRB.**
(DOCX)

Table S2 **Correlation of histologic subtype and expression of 5 cancer associated fibroblast markers.**
(DOCX)

Text S1 **Validation of FAP, FSP1, PDGFRα, and PDGFRβ antibodies.**
(DOCX)
