# Supplementary figures and images for: The Prognostic Significance of Cancer-Associated Fibroblasts in Esophageal Squamous Cell Carcinoma
Source: PLoS One. 2014 Jun 19;9(6):e99955. doi: 10.1371/journal.pone.0099955 (PMC4063790; doi:10.1371/journal.pone.0099955)

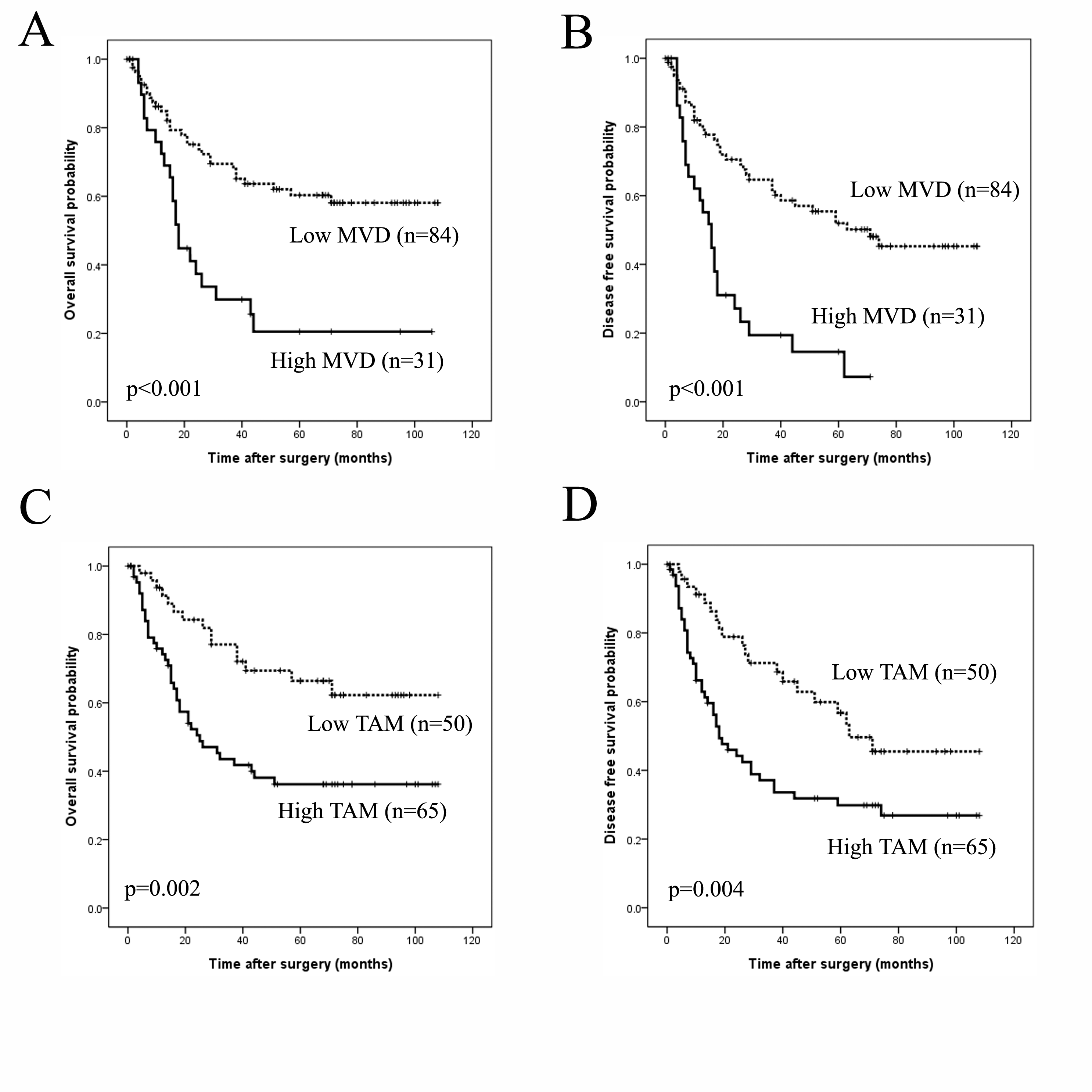

Supplement: Figure S1 — Survival curves using the Kaplan–Meier method by log-rank test for cancer-microenvironment related factors. (A–B) Microvessel density (C–D) Tumor associated macrophages. (TIF) [file pone.0099955.s001.tif]

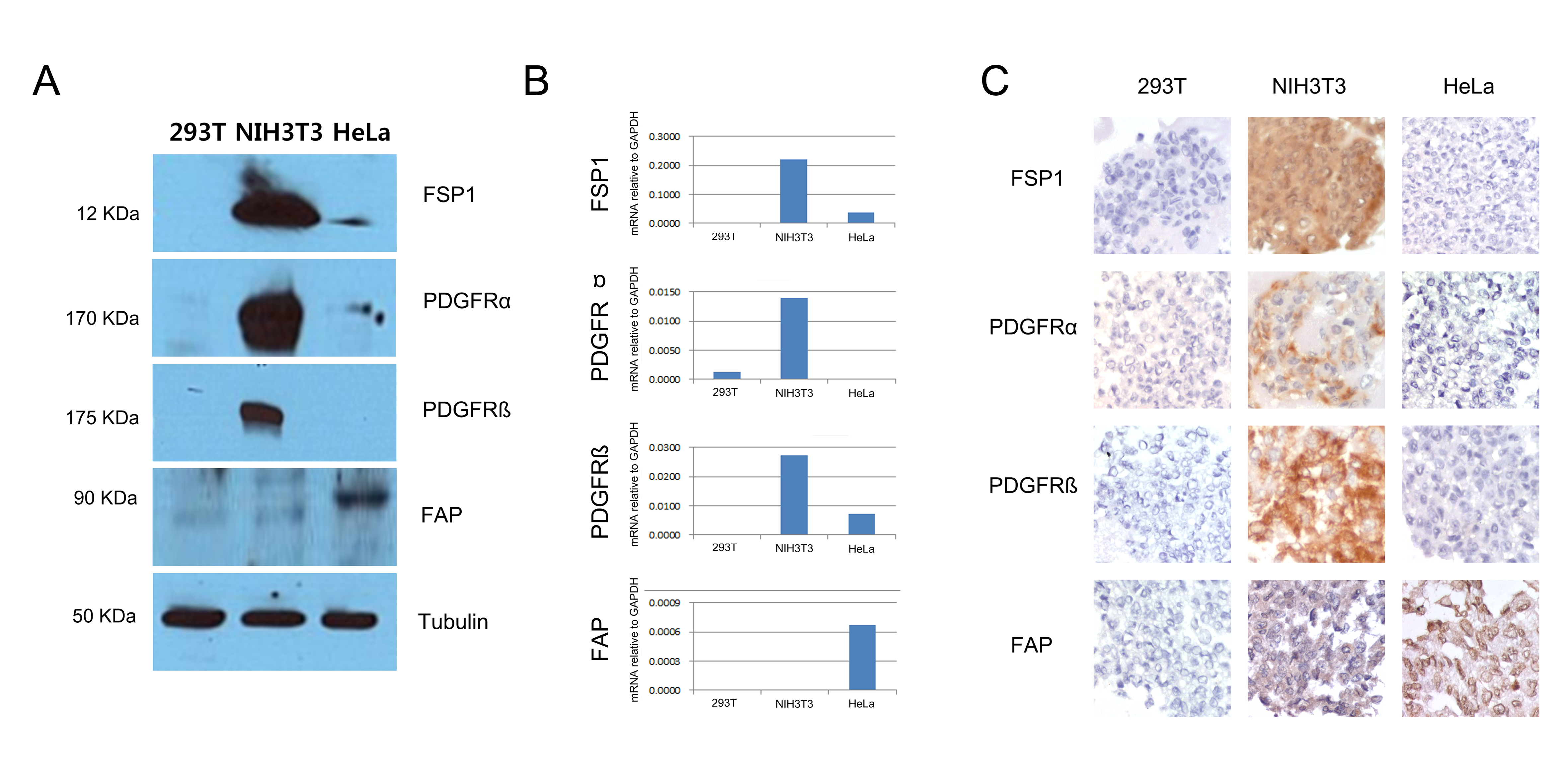

Supplement: Figure S2 — Validation of fibroblast activator protein (FAP), fibroblast-specific protein-1 (FSP-1), platelet-derived growth factor receptor (PDGFR)α and PDGFRβ antibodies. A, B: The results of Western blotting and mRNA level by reverse transcription polymerase chain reaction. All antibodies recognized the proteins with the expected molecular weights (A) and these results were highly consistent with the mRNA levels of these proteins (B). C: The results of immunohistochemical staining of cell blocks from 293T, NIH3T3, and HeLa cells. Antibodies to FSP1, PDGFRα, and PDGFRβ stained in NIH3T3 cells (positive control) but not in 293T and Hela cells (negative control). Antibody to FAP also stained in Hela cells (positive control) but not in 293T or NIH3T3 cells (negative control). (TIF) [file pone.0099955.s002.tif]

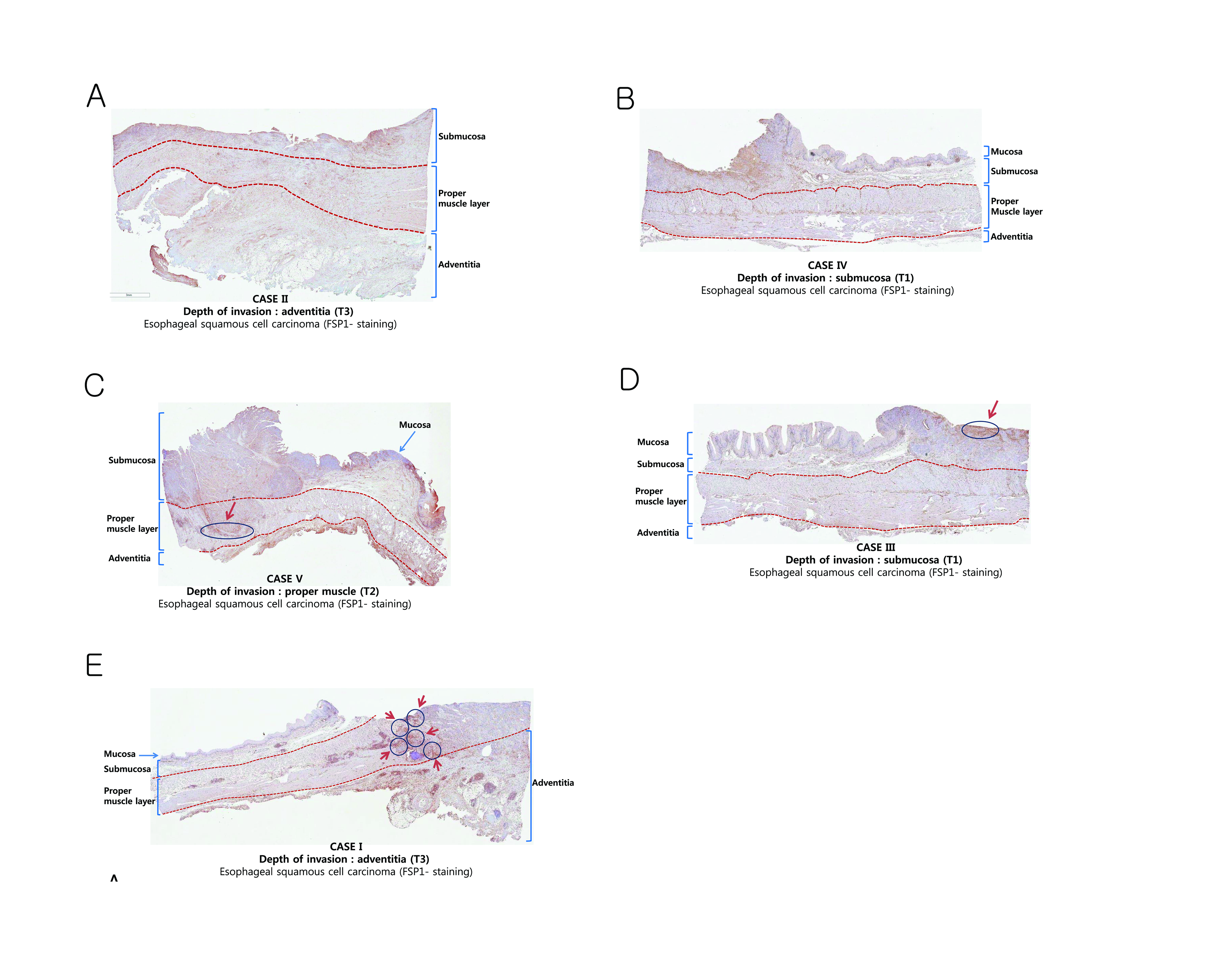

Supplement: Figure S3 — The result of preliminary study examining expression of FSP1, a representative cancer associated fibroblast (CAF) marker, in the whole blocks of 10 esophageal squamous cell carcinoma (ESCC) cases. (A–B) CAFs are evenly distributed irrespective of heterogeneity of CAF phenotype in more than half of ESCC cases. (C–E) The remaining cases show CAF distribution with regional concentration around diverse location, such as invading front (C), surface necrosis (D) or muscle layer (E). (TIF) [file pone.0099955.s003.tif]
