# Supplementary material for: The Prognostic Significance of Cancer-Associated Fibroblasts in Esophageal Squamous Cell Carcinoma
Source: PLoS One. 2014 Jun 19;9(6):e99955. doi: 10.1371/journal.pone.0099955 (PMC4063790; doi:10.1371/journal.pone.0099955)
Supplement: Table S1 — Comparison of clinicopathologic characteristics according to expression pattern of SMA, FSP1, FAP, PDGFRA and PDGRB. (DOCX) [file pone.0099955.s004.docx]

| Table S1. Comparison of clinicopathologic characteristics according to expression pattern of SMA, FSP1, FAP, PDGFRA and PDGRB. | | | | | | | | | | | | | | | | |
| --- | --- | --- | --- | --- | --- | --- | --- | --- | --- | --- | --- | --- | --- | --- | --- | --- |
|  |  |  |  |  |  |  |  |  |  |  |  |  |  |  |  |  |
|  |  | SMA | | | FSP1 | | | FAP | | | PDGFRA | | | PDGFRB | | |
|  | Total | negative | positive | p value | negative | positive | p value | negative | positive | p value | negative | positive | p value | negative | positive | p value |
| n (%) |  | 20 (17.2) | 96 (82.8) |  | 32 (27.6) | 84 (72.4) |  | 45 (38.8) | 71 (61.2) |  | 13 (11.2) | 103 (88.8) |  | 53 (45.7) | 63 (54.3) |  |
| Age (years) |  |  |  |  |  |  |  |  |  |  |  |  |  |  |  |  |
| <65 | 31 (26.7) | 7 (35.0) | 24 (25.0) | 0.358 | 13 (40.6) | 18 (21.4) | 0.037 | 15 (33.3) | 16 (22.5) | 0.200 | 4 (30.8) | 27 (26.2) | 0.744^a^ | 17 (32.1) | 14 (22.2) | 0.232 |
| ≥65 | 85 (73.3) | 13 (65.0) | 72 (75.0) |  | 19 (59.4) | 66 (78.6) |  | 30 (66.7) | 55 (77.5) |  | 9 (69.2) | 76 (73.8) |  | 36 (67.9) | 49 (77.8) |  |
| Gender |  |  |  |  |  |  |  |  |  |  |  |  |  |  |  |  |
| female | 4 (3.4) | 1 (5.0) | 3 (3.1) | 0.536^a^ | 2 (6.3) | 2 (2.4) | 0.305^a^ | 2 (4.4) | 2 (2.8) | 0.641^a^ | 1 (7.7) | 3 (2.9) | 0.383^a^ | 3 (5.7) | 1 (1.6) | 0.330^a^ |
| male | 112 (96.6) | 19 (95.0) | 93 (96.9) |  | 30 (93.8) | 82 (97.6) |  | 43 (95.6) | 69 (97.2) |  | 12 (92.3) | 100 (97.1) |  | 50 (94.3) | 62 (98.4) |  |
| Tumor size (cm) |  |  |  |  |  |  |  |  |  |  |  |  |  |  |  |  |
| <4 | 47 (40.5) | 16 (80.0) | 31 (32.3) | <0.001 | 15 (46.9) | 32 (38.1) | 0.389 | 20 (44.4) | 27 (38.0) | 0.493 | 5 (38.5) | 42 (40.8) | 0.873 | 24 (45.3) | 23 (36.5) | 0.338 |
| ≥4 | 69 (59.5) | 4 (20.0) | 65 (67.7) |  | 17 (53.1) | 52 (61.9) |  | 25 (55.6) | 44 (62.0) |  | 8 (61.5) | 61 (59.2) |  | 29 (54.7) | 40 (63.5) |  |
| Differentiation |  |  |  |  |  |  |  |  |  |  |  |  |  |  |  |  |
| W/D | 17 (14.7) | 11 (55.0) | 6 (6.3) | <0.001^a^ | 5 (15.6) | 12 (14.3) | 0.228^a^ | 4 (8.9) | 13 (18.3) | 0.364 | 2 (15.4) | 15 (14.6) | 1.000^a^ | 9 (17.0) | 8 (12.7) | 0.010 |
| M/D | 76 (65.5) | 6 (30.0) | 70 (72.9) |  | 24 (75.0) | 52 (61.9) |  | 32 (71.1) | 44 (62.0) |  | 9 (69.2) | 67 (65.0) |  | 40 (75.5) | 36 (57.1) |  |
| P/D | 23 (19.8) | 3 (15.0) | 20 (20.8) |  | 3 (9.4) | 20 (23.8) |  | 9 (20.0) | 14 (19.7) |  | 2 (15.4) | 21 (20.4) |  | 4 (7.5) | 19 (30.2) |  |
| T stage |  |  |  |  |  |  |  |  |  |  |  |  |  |  |  |  |
| 1 | 15 (12.9) | 11 (55.0) | 4 (4.2) | <0.001 | 4 (12.5) | 11 (13.1) | 0.315^a^ | 6 (13.3) | 9 (12.7) | 0.886 | 1 (7.7) | 14 (13.6) | 0.738^a^ | 8 (15.1) | 7 (11.1) | 0.881 |
| 2 | 40 (34.5) | 7 (35.0) | 33 (34.4) |  | 12 (37.5) | 28 (33.3) |  | 15 (33.3) | 25 (35.2) |  | 4 (30.8) | 36 (35.0) |  | 17 (32.1) | 23 (36.5) |  |
| 3 | 45 (38.8) | 2 (10.0) | 43 (44.8) |  | 9 (28.1) | 36 (42.9) |  | 19 (42.2) | 26 (36.6) |  | 5 (38.5) | 40 (38.8) |  | 20 (37.7) | 25 (39.7) |  |
| 4 | 16 (13.8) | 0 (0) | 16 (16.7) |  | 7 (21.9) | 9 (10.7) |  | 5 (11.1) | 11 (15.5) |  | 3 (23.1) | 13 (12.6) |  | 8 (15.1) | 8 (12.7) |  |
| N stage |  |  |  |  |  |  |  |  |  |  |  |  |  |  |  |  |
| 0 | 35 (32.1) | 14 (77.8) | 21 (23.1) | <0.001^a^ | 10 (34.50 | 25 (31.3) | 0.587^a^ | 12 (30.0) | 23 (33.3) | 0.336 | 2 (18.2) | 33 (33.7) | 0.146^a^ | 17 (34.7) | 18 (30.0) | 0.284 |
| 1 | 30 (27.5) | 4 (22.2) | 26 (28.6) |  | 7 (24.1) | 23 (28.8) |  | 15 (37.5) | 15 (21.7) |  | 4 (36.4) | 26 (26.5) |  | 10 (20.4) | 20 (33.3) |  |
| 2 | 26 (23.9) | 0 (0) | 26 (28.6) |  | 9 (31.0) | 17 (21.3) |  | 8 (20.0) | 18 (26.1) |  | 5 (45.5) | 21 (21.4) |  | 15 (30.6) | 11 (18.3) |  |
| 3 | 18 (16.5) | 0 (0) | 18 (19.8) |  | 3 (10.3) | 15 (18.8) |  | 5 (12.5) | 13 (18.8) |  | 0 (0) | 18 (18.4) |  | 7 (14.3) | 11 (18.3) |  |
| M stage^b^ |  |  |  |  |  |  |  |  |  |  |  |  |  |  |  |  |
| 0 | 99 (85.3) | 19 (95.0) | 80 (83.3) | 0.299^a^ | 25 (78.1) | 74 (88.1) | 0.239^a^ | 39 (86.7) | 60 (84.5) | 0.749 | 10 (76.9) | 89 (86.4) | 0.403^a^ | 44 (83.0) | 55 (87.3) | 0.516 |
| 1 | 17 (14.7) | 1 (5.0) | 16 (16.7) |  | 7 (21.9) | 10 (11.9) |  | 6 (13.3) | 11 (15.5) |  | 3 (23.1) | 14 (13.6) |  | 9 (17.0) | 8 (12.7) |  |
| Microvessel density^c^ |  |  |  |  |  |  |  |  |  |  |  |  |  |  |  |  |
| low | 45 (39.1) | 5 (25.0) | 40 (42.1) | 0.210 | 14 (43.8) | 31 (37.3) | 0.82 | 17 (37.8) | 28 (40.0) | 0.092 | 9 (69.2) | 36 (35.3) | 0.059^a^ | 23 (43.4) | 22 (35.5) | 0.488 |
| intermediate | 39 (33.9) | 10 (50.0) | 29 (30.5) |  | 10 (31.3) | 29 (34.9) |  | 20 (44.4) | 19 (27.1) |  | 3 (23.1) | 36 (35.3) |  | 15 (28.3) | 24 (38.7) |  |
| high | 31 (27.0) | 5 (25.0) | 26 (27.4) |  | 8 (25.0) | 23 (27.7) |  | 8 (17.8) | 23 (32.9) |  | 1 (7.7) | 30 (29.4) |  | 15 (28.3) | 16 (25.8) |  |
| Tumor associated macrophages^c^ |  |  |  |  |  |  |  |  |  |  |  |  |  |  |  |  |
| low | 50 (43.5) | 11 (55.0) | 39 (41.1) | 0.253 | 14 (43.8) | 36 (43.4) | 0.971 | 22 (50.0) | 28 (39.4) | 0.267 | 5 (41.7) | 45 (43.7) | 0.894 | 22 (42.3) | 28 (44.4) | 0.818 |
| high | 65 (56.5) | 9 (45.0) | 56 (58.9) |  | 18 (56.3) | 47 (56.6) |  | 22 (50.0) | 43 (60.6) |  | 7 (58.3) | 58 (56.3) |  | 30 (57.7) | 35 (55.6) |  |
| Epthelial to mesenchymal transition |  |  |  |  |  |  |  |  |  |  |  |  |  |  |  |  |
| complete | 25 (21.6) | 1 (5.0) | 24 (25.0) | 0.005^a^ | 6 (18.8) | 19 (22.6) | 0.514 | 10 (22.2) | 15 (21.1) | 0.907 | 1 (7.7) | 24 (23.3) | 0.207^a^ | 11 (20.8) | 14 (22.2) | 0.830 |
| incomplete | 31 (26.7) | 2 (10.0) | 29 (30.2) |  | 11 (34.4) | 20 (23.8) |  | 11 (24.4) | 20 (28.2) |  | 6 (46.2) | 25 (24.3) |  | 13 (24.5) | 18 (28.6) |  |
| wild | 60 (51.7) | 17 (85.0) | 43 (44.8) |  | 15 (46.9) | 45 (53.6) |  | 24 (53.3) | 36 (50.7) |  | 6 (46.2) | 54 (52.4) |  | 29 (54.7) | 31 (49.2) |  |
| ^a^ by Fisher's exact test, otherwise chi square test | | | | | | | | | | | | | | | | |
| ^b^ Severn cases of unsatisfactory for minimal number of evaluated lymph nodes, were excluded in the analysis. | | | | | | | | | | | | | | | | |
| ^c^ One case was excluded in the analysis of microvessel density and macrophages due to lack of tissue. | | | | | | | | | | | | | | | | |
|  | | | | | | | | | | | | | | | | |
|  | | | | | | | | | | | | | | | | |
