# Supplementary material for: The Prognostic Significance of Cancer-Associated Fibroblasts in Esophageal Squamous Cell Carcinoma
Source: PLoS One. 2014 Jun 19;9(6):e99955. doi: 10.1371/journal.pone.0099955 (PMC4063790; doi:10.1371/journal.pone.0099955)
Supplement: Table S2 — Correlation of histologic subtype and expression of 5 cancer associated fibroblast markers. (DOCX) [file pone.0099955.s005.docx]

| Table S2. Correlation of histologic subtype and expression of 5 cancer associated fibroblast markers. | | | | | |
| --- | --- | --- | --- | --- | --- |
|  |  |  |  |  |  |
|  | SMA | FSP1 | FAP | PDGFRα | PDGFRß |
| Mature | 45/52 (86.5%) | 32/52 (61.5%) | 28/52 (53.8%) | 41/52 (78.8%) | 22/52 (42.3%) |
| Immature | 51/64 (79.7%) | 52/64 (81.3%) | 43/64 (67.2%) | 62/64 (96.9%) | 41/64 (64.1%) |
| p value | 0.331 | 0.018 | 0.143 | 0.002 | 0.019 |
