# Supplementary material for: The Prognostic Significance of Cancer-Associated Fibroblasts in Esophageal Squamous Cell Carcinoma
Source: PLoS One. 2014 Jun 19;9(6):e99955. doi: 10.1371/journal.pone.0099955 (PMC4063790; doi:10.1371/journal.pone.0099955)
Supplement: Text S1 — Validation of FAP, FSP1, PDGFRα, and PDGFRβ antibodies. (DOCX) [file pone.0099955.s006.docx]

**Text S1. Validation of FAP, FSP1, PDGFRα, and PDGFRß antibodies**

**Materials and Methods**

**Cell lines**

The 293T, NIH3T3, and Hela cell lines were maintained in Dulbecco’s modified Eagle’s medium with a high concentration of glucose (Life Technologies, Grand Island, NY, USA) supplemented with 10% heat-inactivated fetal bovine serum (Life Technologies), 100 mg/mL penicillin G, and 50 μg/mL streptomycin (Life Technologies) at 37°C in a humidified atmosphere containing 5% CO_2_. All cell lines were purchased from the Korean Cell Line Bank (Seoul, Korea).

**RNA extraction and quantitative real-time reverse transcription polymerase chain reaction (RT-PCR)**

Total RNA was extracted using a standard phenol–chloroform extraction and isopropanol precipitation method. Complementary DNA (cDNA) was synthesized from 2 μg total RNA using Superscript II RNA Reverse Transcriptase (#11904-018; Invitrogen, Carlsbad, CA, USA), according to the manufacturer’s protocol. Real-time RT-PCR was performed using the ABI 7900 HT Fast real-time PCR system (Applied Biosystems, Foster City, CA, USA). SYBR Green PCR Master Mix (Applied Biosystems) was employed for the PCR. The primers used in this study were as follows:

| FAP: F: ACGGCTTATCACCTGATCGG |
| --- |
| R: AATTGGACGAGGAAGCTCATTT  FSP1: F: GATGAGCAACTTGGACAGCAA  R: CTGGGCTGCTTATCTGGGAAG |
| PDGFRá: F:AACCGTGTATAAGTCAGGGGA |
| R:GCATTGTGATGCCTTTGCCTT  PDGFRß: F: TGGG CCTCTGTGGG GGCTGCTGGC  R: AGCCAATGAGGGGTCCAAGCCGA  GAPDH: F:GCACCGTCAAGGCTGAGAA  R:AGCATCGCCCCACTTGATT |

Glyceraldehyde 3-phosphate dehydrogenase was used as an internal loading control.

**Western blotting analysis**

Cell lysates were prepared using an sodium dodecyl sulfate (SDS) lysis solution, and protein concentration was measured using a bicinchoninic acid protein assay kit (Pierce, Rockford, IL, USA). Equal amounts of protein were separated by electrophoresis on 12.5% SDS-polyacrylamide gel electrophoresis. The proteins were electrotransferred from the gel to a nitrocellulose membrane, which was blocked with a 5% nonfat milk solution for 1 hour and then incubated with primary anti-FAP (1:1000, Abcam, Cambridge, UK), anti-FSP1 (1:1000, Millipore, MA, USA), anti-PDGFRα (1:1000, Cell Signaling Technology, Beverley, MA, USA) and anti-PDGFRß (1:1000, Abcam) for 2 hours at room temperature. Anti-tubulin antibody (1:1000; Santa Cruz Biotechnology, Santa Cruz, CA, USA) was used as an internal loading control. The membrane was incubated with goat anti-rabbit secondary antibodies that were detected with an enhanced chemiluminescence detection system (Amersham Biosciences, Freiburg, Germany) according to the manufacturer’s instructions.

**Preparation of cell blocks for antibody validation**

Cells were trypsinized, fixed for 1 hr in 10 % formalin, and then centrifuged and resuspended in 0.8% agarose. The gel plugs containing fixed tumor cells were then processed through standard pathology specimen processing proceducre. Once processed, cells were embedded in paraffin and then used for antibody validation.

**Result**

**Validation of FAP, FSP1, PDGFRα, and PDGFRß antibodies**

We used three cell lines such as 293T, NIH3T3, Hela as positive or negative controls to validate the antibodies recognizing FAP, FSP1, PDGFRα, and PDGFRß. First, we examined the specificity of these antibodies using Western blotting and real-time RT-PCR. All of the antibodies recognized the proteins with expected molecular weights (Figure 3A), and these results were highly consistent with mRNA levels of these proteins assessed by real time RT-PCR (Figure 3B). For further validation, NIH3T3 cells were used as a positive control for FSP-1, PDGFRα, and PDGFRß, and Hela cells were used as a positive control for FAP. To validate the specificity of these antibodies in immunostaining of formalin fixed and paraffin embedded tissues, these cell lines were fixed in formalin and embedded in paraffin following the standard pathology specimen processing procedure. Then we immunostained the tissue sections from these cell blocks with those antibodies. Antibodies to FSP1, PDGFRα, and PDGFRß stained NIH3T3 cells (positive control) but did not stain 293T or Hela cells (negative control) (Figure 3C). The FAP antibody also stained Hela cells (positive control) but not 293T or NIH3T3 cells (negative control) (Figure 3C). Thus, we concluded that all these antibodies were specific for immunostaining formalin fixed and paraffin embedded tissues.
